# A Case of Extensive Pneumocephalus Following an Electric Scooter Accident

**DOI:** 10.7759/cureus.90361

**Published:** 2025-08-18

**Authors:** Akihiro Kato, Kazunori Imai, Miona Komatsu, Akito Kubota, Asako Matsushima

**Affiliations:** 1 Department of Emergency and Critical Care, Nagoya City University, Graduate School of Medical Sciences, Nagoya, JPN; 2 Anesthesia and Intensive Care, Nagoya City University East Medical Center, Nagoya, JPN

**Keywords:** airway management, electric scooter injury, facial trauma, pneumocephalus, traumatic brain injury

## Abstract

The use of electric scooters (e-scooters) has rapidly increased in Japan, accompanied by a rise in associated injuries. Severe head trauma can occur even when the Glasgow Coma Scale score suggests a mild injury, particularly when major clinical signs are present. Therefore, careful observation and appropriate management are essential. A 42-year-old woman injured by an e-scooter accident was transferred to our hospital with facial swelling and right vision loss. Head computed tomography revealed extensive pneumocephalus, skull base fractures, and traumatic subarachnoid hemorrhage. She subsequently developed status epilepticus, requiring rapid sequence intubation and deep sedation. Conservative management was adopted, and the pneumocephalus and a suspected cerebrospinal fluid leak resolved by day 8. The patient was discharged on day 21 without a consciousness disturbance. E-scooter-related trauma may conceal severe head trauma, even when initial neurological assessment suggests a mild injury. This case may suggest that prompt assessment and timely airway management are essential to prevent secondary brain injury in patients with extensive pneumocephalus.

## Introduction

Electric scooters (e-scooters) have recently gained popularity in Japan, particularly in urban areas, due to their convenience and maneuverability. However, the surge in usage has been accompanied by a notable increase in serious injuries. In several countries, e-scooter-related trauma has become a public health concern, prompting discussions on helmet legislation and traffic regulation. A trauma registry from France reported a 25.9% incidence of severe traumatic brain injury and a 9.2% mortality rate associated with e-scooter accidents, figures that exceed those reported for motorcycle crashes [1]. Similarly, a retrospective study from a metropolitan Level I trauma center reported a sixfold increase in e-scooter-related emergency visits - many requiring surgery or hospitalization - after the launch of shared e-scooter services [2].

Among the spectrum of injuries sustained, facial fractures can extend to the skull base, posing a risk for complications such as pneumocephalus or, in rare cases, life-threatening tension pneumocephalus. These conditions necessitate prompt recognition, careful airway management, and appropriate intensive care to mitigate the risk of elevated intracranial pressure [3]. In Japan, detailed clinical reports concerning severe trauma related to electric scooters remain scarce. We herein present a rare case of extensive pneumocephalus following an e-scooter accident that was successfully treated by conservative management.

## Case presentation

A 42-year-old woman (156 cm, 39 kg) with no significant prior medical history fell while riding an electric scooter after swerving to avoid a pedestrian, striking the right side of her face. She was not wearing a helmet. She experienced visual disturbance and epistaxis and was transported to our emergency department.

On arrival, vital signs were: Glasgow Coma Scale (GCS) was E4V5M6, respiratory rate was 13 breaths/min, heart rate was 64 beats/min, blood pressure was 151/90 mmHg, and oxygen saturation was 100% on room air. The right pupil measured 5 mm and was non-reactive, while the left was 4 mm and reactive. No light perception was noted in the right eye. Although facial swelling was evident, there was no active bleeding or deformity. No other life-threatening injuries were observed.

**Figure 1:**
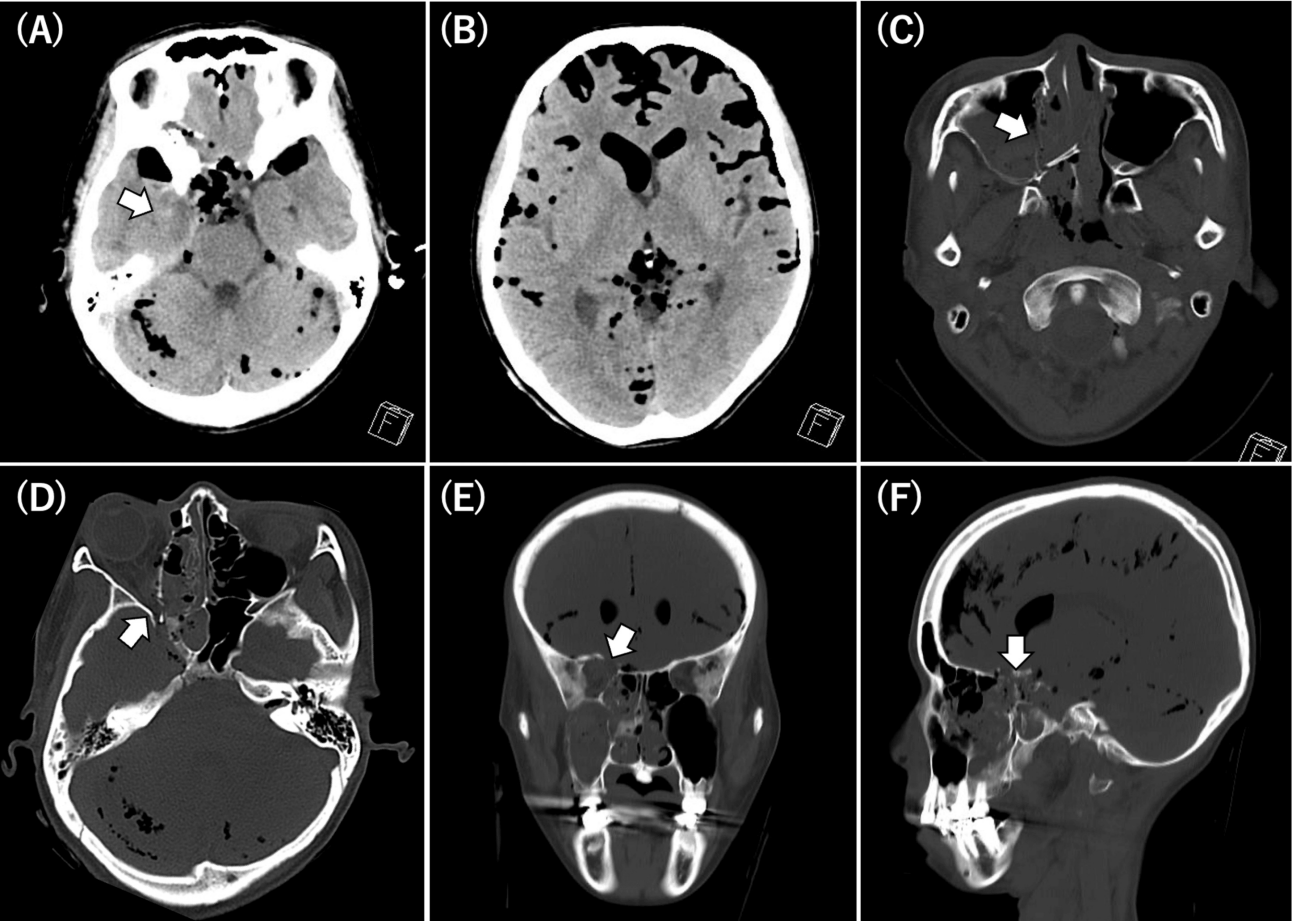
Head CT on admission. A: Right temporal lobe contusion (arrow). B: Extensive pneumocephalus along the cerebral sulci. C: Fractures from the maxillary sinus wall to the nasal septum(arrow). D: Optic canal fracture (arrow). E: Fractures of the orbital roof and sphenoid bone are observed in the coronal view (arrow). F: Fractures of the orbital roof and sphenoid bone are visualized in the sagittal view (arrow).

Head computed tomography revealed fractures involving the right orbital roof, skull base (including the sphenoid sinus), optic canal, nasal septum, and maxillary sinus wall. Associated intracranial injuries included a right temporal lobe contusion, traumatic subarachnoid hemorrhage, and extensive pneumocephalus (Figure 1). As the airway was stable and there were no signs of respiratory distress, emergent airway intervention was initially deferred.

While preparing for intensive care unit admission, she developed status epilepticus. Although the seizures ceased after the administration of intravenous diazepam (5 mg), they were followed by bradypnea. Minimal positive pressure ventilation was provided using a Jackson-Rees circuit. Due to concern about the possible exacerbation of pneumocephalus, rapid sequence induction was performed using propofol (50 mg) and rocuronium (50 mg), followed by the initiation of mechanical ventilation in pressure-controlled mode (PEEP 3 cmH₂O, inspiratory pressure 7 cmH₂O).

Nasal endoscopy revealed no cerebrospinal fluid leakage, and conservative management was adopted. In the intensive care unit, sedation with propofol and fentanyl, along with continuous rocuronium infusion, was administered. Levetiracetam (1000 mg/day) and ceftriaxone (2 g/day) were initiated.

**Figure 2:**
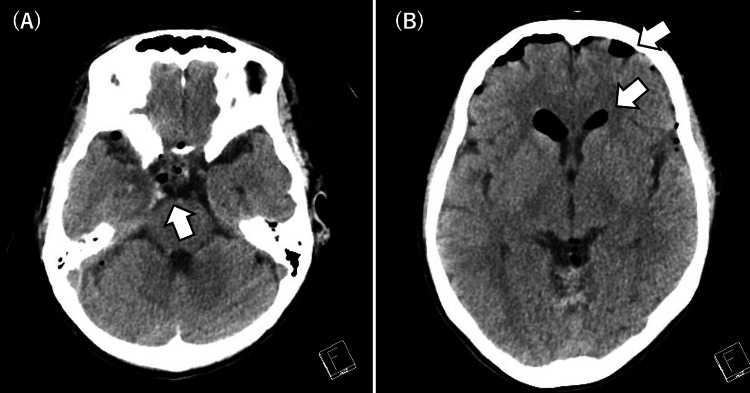
Head CT on post-injury day 1. A: Decreased air in the suprasellar cistern (arrow). B: Decreased air in the ventricles and on the cortical surface of the brain (arrow).

Repeat computed tomography on day 2 demonstrated improvement in the patient’s pneumocephalus (Figure 2). Sedation was discontinued on day 3, and the patient was successfully extubated. By day 8, the pneumocephalus and suspected cerebrospinal fluid leak had resolved. Lacosamide (50 mg twice daily) was added on day 9. No further seizures occurred. Although right-sided visual loss persisted, the patient was discharged from the hospital on day 21 without a consciousness disturbance.

## Discussion

Head trauma is common in e-scooter accidents. A French multicenter study reported head involvement in 38.4% of patients with Abbreviated Injury Scale ≥3 injuries [1]. Risk factors include small body size, high speed, and alcohol use [1,4]. In this case, the patient’s low body mass index (16) might have contributed to the severe head and facial injury. Although helmet use is effective for avoiding severe head injury, helmet usage rates among e-scooter users remain low (1.1-22.5%) [1,5,6]. In Japan, riders of ≥16 years of age can operate e-scooters at speeds of up to 20 km/h without a license, and helmet use is encouraged but not mandatory [7]. In the present case, the patient was not wearing a helmet at the time of the accident. The combination of these factors could have resulted in severe head trauma.

In this case, initial head computed tomography showed fractures from the maxillary sinus to the nasal septum, and involving the orbital roof, sphenoid bone, and optic canal, suggesting a high-energy frontal or anterolateral impact. The force likely propagated to the skull base, causing extensive pneumocephalus via communication between the paranasal sinuses and the intracranial cavity through a sphenoid fracture. Effective conservative care for extensive pneumocephalus requires minimizing intracranial-extracranial communication and stabilization of the intracranial pressure. The strong inspiratory effort associated with sneezing and coughing can increase intracranial air and trigger tension pneumocephalus [8,9]. Our patient developed status epilepticus, requiring rapid sequence intubation. We speculate that early rapid sequence intubation and neuromuscular blockade to suppress spontaneous breathing enabled blockade of the intracranial-extracranial communication, which contributed to stabilization of the intracranial pressure and improved the pneumocephalus. Regarding traumatic optic neuropathy, conservative management was chosen because the efficacy of high-dose steroids or optic canal decompression  has not been established [10-12]. The absence of a visible fistula on nasal endoscopy supported nonoperative treatment for the fractures of the anterior skull base, including the optic canal.

## Conclusions

This case demonstrated that electric scooter accidents can cause severe head injuries, even when initial neurological assessment suggests a mild injury. This case suggests that in patients with extensive pneumocephalus, conservative management with rapid sequence intubation and neuromuscular blockade to suppress spontaneous breathing might contribute to stabilization of intracranial pressure and improvement of the pneumocephalus.
